# Health Outcome Determinants of Human Papillomavirus Vaccination in Adult Women in Spain

**DOI:** 10.3390/vaccines14050460

**Published:** 2026-05-21

**Authors:** Jesús de la Fuente-Valero, Javier Rejas-Gutiérrez, Marta del Pino, Carmen González-Granados, Raquel Oliva-Sánchez, Beatriz Procas-Ramón, Mar Ramírez-Mena, Aaron Cohen-Castiel, Javier Calvo-Torres, María Fasero, Pluvio J. Coronado, on behalf of the HPV-Know Collaborative Group, SPAIN-GOG

**Affiliations:** 1 Department of Obstetrics and Gynecology, Hospital Universitario Infanta Leonor, 28031 Madrid, Spain; jesus.fuente@salud.madrid.org; 2 EACCOS Research Group, School of Psychology, Universidad Autónoma de Madrid, 28049 Madrid, Spain; rejasjavier1958@gmail.com; 3 Institute Clinic of Gynecology, Obstetrics and Neonatology, Hospital Clínic, Universitat de Barcelona, 08036 Barcelona, Spain; 4 Department of Obstetrics and Gynecology, Hospital de la Axarquía, 29700 Málaga, Spain; 5 Department of Obstetrics and Gynecology, Hospital Virgen de la Arrixaca, 30120 Murcia, Spain; 6 Department of Obstetrics and Gynecology, Hospital General Universitario San Jorge, 22004 Huesca, Spain; 7 Women’s Health Institute, Hospital Clínico San Carlos (IdISSC), 28040 Madrid, Spain; 8 Clínica Dexeus Mujer, Hospital Universitari Dexeus, 08028 Barcelona, Spain; 9 Clinica Corofas Menopause, 13700 Tomelloso, Spain; 10 School of Medicine, Universidad Francisco de Vitoria, 28223 Madrid, Spain; 11 School of Medicine, Universidad Complutense de Madrid, 28040 Madrid, Spain

**Keywords:** human papillomavirus (HPV), knowledge, adult women, nationwide study, vaccination determinants, HPV infection, quality of life

## Abstract

**Background/Objectives:** Health outcome determinants affecting Human Papillomavirus (HPV) vaccination among the adult female population are scarce in Spain. This study aimed to describe the health outcomes and determinants of HPV vaccination in women 18–65 years attending lower genital tract outpatient clinics across regions of Spain. **Methods:** This was a cross-sectional, multicenter, non-interventional, descriptive, and comparative nationwide study. Sociodemographic characteristics and health outcomes included obstetric, gynecological and HPV vaccination antecedents, together with patient-reported outcomes related to HPV infection. Statistical analysis included multivariate logistic regression models. **Results:** Among 2004 adult women recruited, 1907 (95.2%) were eligible for analysis. Vaccine uptake was 48.8%; 81.6% among women who were ever HPV positive (adjusted OR = 2.16 [95% CI: 1.59–2.93], *p* < 0.001), but 65.9% among women with an active infection, which acted as a negative factor for vaccination (OR = 0.63 [0.45–0.87], *p* = 0.005), as did increasing age (OR = 0.92 [0.90–0.93], *p* < 0.001); the higher the age, the lower the adjusted likelihood of being vaccinated. HPV knowledge and adequate physician-provided information were weakly associated with vaccination likelihood. A history of conization (OR = 7.48 [5.34–10.47], *p* < 0.001), use of contraception (OR = 1.49 [1.13–1.96], *p* = 0.004), infection with high-risk or unknown-risk HPV genotypes (OR = 1.86 [1.23–2.82], *p* = 0.003 and OR = 1.68 [1.17–2.42], *p* = 0.006, respectively), and Spanish nationality (OR = 2.46 [1.68–3.61], *p* < 0.001) were identified as factors associated with a higher vaccination likelihood. **Conclusions:** This study found that HPV vaccination uptake is improvable. Previous HPV infection favored vaccination; however, active infection and increasing age acted against vaccination. HPV knowledge and adequate healthcare professional information appeared to favor vaccination, along with, most notably, a history of cervical surgery (conization), contraceptive use, or infection with high-risk or unknown-risk HPV genotypes. Spanish women had a higher likelihood of receiving HPV vaccination than foreign residents.

## 1. Introduction

Human papillomavirus (HPV) is one of the most common sexually transmitted infections (STI) worldwide and is widely recognized as the primary etiologic agent in the development of pre-neoplastic lesions and cancers of the anogenital tract, as well as head and neck regions [1,2,3]. Likewise, it is well established that a HPV diagnosis has a significant impact on women, not only physically due to the lesions it causes, but also sexually, socially, and emotionally [4,5].

This can lead to deterioration in emotional relationships with partners, exacerbation of existing sexual problems, and a decline in health-related quality-of-life (HRQoL) [6,7]. In addition to the distress caused by HPV, women may experience anxiety related to subsequent gynecological tests and treatments, which can reduce adherence to follow-up recommendations. This suggests that appropriate counseling and a clear understanding of HPV-related diseases could improve women’s psychological responses and health outcomes [8].

Real-world data on prophylactic HPV vaccination have demonstrated high effectiveness in preventing HPV infection, HPV-related cervical precancerous lesions, and cervical cancer when administered up to 30 years of age [9,10,11,12].

Studies conducted in adolescent populations have shown that HPV vaccination does not alter sexual behavior, does not increase risky sexual activity, and does not negatively affect sexual relationships [13].

In contrast, few studies have examined the impact of HPV vaccination on the sexual and reproductive health of adult women. One study reported that some adult women (13%) felt that being vaccinated against HPV increased their sense of confidence or safety in their relationships, as HPV was perceived as “one less thing” to worry about [14].

In Spain, HPV vaccination for adult women is included in the national vaccination program under several contexts, including catch-up vaccination and risk-based indications [15]. The COVAR Study estimated an annual HPV vaccination coverage rate (VCR) of 87.3% among women undergoing conization for SIL/CIN [16]. However, among Spanish women aged 15–55 years, who were not included in the national vaccination program, cumulative HPV vaccination coverage between 2007 and 2020 was approximately 4.03% [17]. Despite growing interest in HPV vaccination beyond adolescence, evidence on vaccination uptake and its determinants in adult women remains limited, particularly in real-world clinical settings.

Numerous factors may influence vaccine coverage and effectiveness, including age at vaccination, geographic region, and level of education [18,19,20]. Other determinants affecting vaccination uptake in women are not yet well established [21]. A lack of awareness or access to accurate information about HPV is one of the key reasons why primary prevention measures are not fully implemented [22,23,24]. Studies have shown that HPV knowledge in Europe is moderate but varies considerably across countries and populations [25].

The clinical profile and health outcomes of vaccinated versus unvaccinated adult women are not well characterized, and the role of clinical and health-related factors in influencing HPV vaccination uptake in adult women remains poorly understood, particularly in the Spanish context.

Therefore, given the lack of comprehensive data on the determinants of HPV vaccination and associated health outcomes among Spanish-speaking adult women, this study aims to identify factors associated with HPV vaccination compared with non-vaccination in adult women in Spain. In addition, a descriptive analysis of sociodemographic characteristics, clinical history, and patient-reported outcomes (PROs) in vaccinated adult women is undertaken.

This knowledge could support public health policies, inform clinical practice guidelines, and help reduce inequalities in the prevention of HPV-related diseases. Ultimately, increasing HPV vaccination coverage among adult women could further mitigate the physical, sexual, social, and emotional burden associated with HPV-related conditions.

## 2. Materials and Methods

### 2.1. Study Design and Setting

This was a cross-sectional, multicenter, non-interventional, descriptive, and comparative nationwide study, conducted in a representative sample of Spanish adult women between January 2022 and December 2024. Participants were recruited from gynecology and lower genital tract outpatient clinics in twenty-four public and private centers across all regions of Spain. This study was not sponsored by any institution, healthcare provider, or pharmaceutical company. The authors developed the study concept and design. The protocol was approved by the Clinical Research Ethics Committee of Hospital Clínico San Carlos, Universidad Complutense de Madrid (approval date: 10 September 2020; code 20/590-E), and was conducted in accordance with the principles of the Declaration of Helsinki and the current applicable Good Clinical Practice guidelines of the International Council for Harmonization. All patients provided written informed consent, and the study was carried out under routine clinical practice. This study adhered to STROBE guidelines [26]. Each participant completed a case report form collecting sociodemographic information, HPV vaccination status, obstetric and gynecological history, including possible HPV infection, knowledge about HPV, and several patient-reported outcome measures (PROMs). Participant investigators and authors exclusively collected, analyzed, and interpreted the recorded data.

### 2.2. Study Participants and Sample Size Determination

In addition to HPV vaccination status, eligible women were Spanish-speaking residents in Spain, aged 18 to 65 years, who had provided written informed consent and were consecutively attending gynecology and lower genital tract outpatient clinics. Pregnant women, those with cancer undergoing recent treatment, those with severe psychiatric illness, those who refused to provide written informed consent, and those not fluent in Spanish were excluded from the study. The sample size was determined based on an expected HPV vaccination uptake of 40% among participants [25,27]. The confidence level was set at 95%. With a precision of 2% and a 10% increase in sample size to account for potential missing data, this study aimed to recruit 1760 women aged 18–65 years in order to include at least 863 women who had been vaccinated against HPV. Non-vaccinated women constituted the comparison group.

### 2.3. Clinical Assessments and PROs

All participants provided sociodemographic data, HPV infection and vaccination antecedents, HPV type (low- or high-risk type and genotype, if available), and obstetric and gynecological history, including information from the most recent cytology examination. HPV infection status was differentiated into infection at any time (infection prior to the study visit) and active infection at the time of the study visit. For vaccinated women, vaccination included the type of vaccine, number of doses received, and time elapsed since vaccination. Information regarding recurrent HPV infection, HPV genotype, and cytology findings after vaccination was recorded when available. Participants also completed a questionnaire assessing their knowledge about HPV (HPV-Know-Q) in its validated Spanish version [28]. Women with a HPV-positive infection were additionally invited to complete the following patient-reported outcome measures (PROMs): the Hospital Anxiety and Depression Scale (HADS), the 12-item General Health Questionnaire (GHQ-12) by Goldberg et al., the Female Sexual Function Index (FSFI) by Rosen et al., the specific HPV quality-of-life questionnaire (HPV-QoL), and the aforementioned HPV-Know-Q, all in their validated Spanish versions [28,29,30,31,32].

Participant HPV knowledge was assessed using the recently validated and innovative HPV-Know-Q questionnaire. This instrument includes two subtests addressing key aspects of HPV infection, including its impact on both sexes, transmission, progression, associated diseases, prevention, and vaccination. One subtest consists of 10 multiple-choice questions with three possible answers (only one correct), while the other consists of 10 true/false questions. Each correct answer contributes 10 points to the total score, with no penalties for incorrect responses. The maximum score for each subtest is 100 points, and the overall knowledge score is calculated as the average of the two subtests. The HADS is a 14-item self-administered scale assessing symptoms of anxiety and depression, with seven items per domain. Each item is scored from 0 to 3. Domain scores range from 0 to 21 and are categorized into four severity groups: normal (0–7), mild (8–10), moderate (11–14), and severe (15–21). The GHQ-12 is a questionnaire used to assess self-perceived mental health. It consists of 12 items scored on a Likert-type scale from 0 to 3, grouped into three domains: self-esteem, stress, and coping. The total score ranges from 0 to 36, reflecting a continuum from no mental impairment to significant psychological distress. A score of 17 or higher indicates mental distress. The FSFI is a multidimensional self-report instrument assessing female sexual function over the previous four weeks. It comprises 19 items across six domains: sexual desire, arousal, lubrication, orgasm, satisfaction, and pain. Total scores range from 2 to 36, with higher scores indicating better sexual function. Sexual dysfunction was defined as a composite score < 26.55 points. The HPV-QoL questionnaire is a self-administered, multidimensional, and innovative patient-reported tool that evaluates the impact of HPV infection on daily life, general health, feelings, and behaviors. It includes 15 items across four domains (one with two subdomains): general wellbeing (including psychological and social wellbeing), sexuality, health, and contagiousness. The scale provides a standardized score ranging from 0 (worst quality of life) to 100 (best quality of life).

### 2.4. Endpoints

As mentioned, this cross-sectional study was conducted to investigate health outcome factors related to HPV vaccination in adult women attending gynecology and lower genital tract outpatient clinics in Spain as the primary objective. In addition to sociodemographic data, the health outcomes evaluated included obstetric and gynecological history, HPV vaccination antecedents, and patient-reported outcomes that were initially considered to be associated with HPV vaccination and/or HPV infection. Secondarily, the study examined vaccination-related health outcomes in the subsample of women infected with HPV.

### 2.5. Statistical Analysis

First, the statistical analysis involved validation of the dataset, including identification of potential recording errors, variables with missing data, and calculation of the rate and type of missing data. Missing data imputation was performed using different methods depending on the variable and type of missingness. Missing items in PROMs were handled according to the scoring rules of each questionnaire or by predictive algorithms using regression modeling. Missing sociodemographic data were imputed using the overall mean or the most frequent response for each variable when missing data were present in less than 10% of participants. Supplementary Materials Table S9 includes missing data percentages for each variable in the study. Qualitative variables are presented as absolute frequencies and relative percentages, whereas quantitative variables are presented as means, standard deviations (SD), and 95% confidence intervals (CI), when appropriate. The Kolmogorov–Smirnov test was used to assess the normality of key variable distributions. Univariate analyses were conducted to compare sociodemographic and clinical variables, antecedents, and PROMs. Parametric tests (Student’s *t*-test or ANOVA) were used for normally distributed data, and non-parametric tests (Mann–Whitney or Kruskal–Wallis) for non-normally distributed data. Welch and Brown–Forsythe robust tests were applied for comparisons of means when homogeneity of variance was not met, as assessed by Levene’s test. Categorical variables were compared using contingency tables and the chi-square (χ^2^) test or Fisher’s exact test.

Determinants of health outcomes associated with HPV vaccination, both in the overall sample and in the HPV-positive subsample, were identified using multivariate binary logistic regression models (with HPV vaccination as the dependent variable), applying a backward stepwise procedure and using the Wald statistic to retain significant explanatory variables. Covariates identified in univariate analyses with *p* < 0.05 were included in the models. Odds ratios (ORs) and 95% confidence intervals were calculated. A similar regression model, including age at vaccination and number of vaccine doses as covariates and categorical multilevel variables as dummy variables, was applied to estimate the probability of recurrent HPV infection after vaccination according to the type of vaccine administered. Statistical significance was set at *p* < 0.05. For statistically significant results, effect sizes were calculated using Cohen’s d and were interpreted as small (0.2 < d < 0.50), moderate (0.50 < d < 0.80), or large (d > 0.80) [33]. Statistical analyses were performed using IBM SPSS version 26.0 (Armonk, NY, USA statistical package (https://www.ibm.com/analytics/spss-statistics-software, accessed on 18 December 2025).

## 3. Results

**Figure 1:**
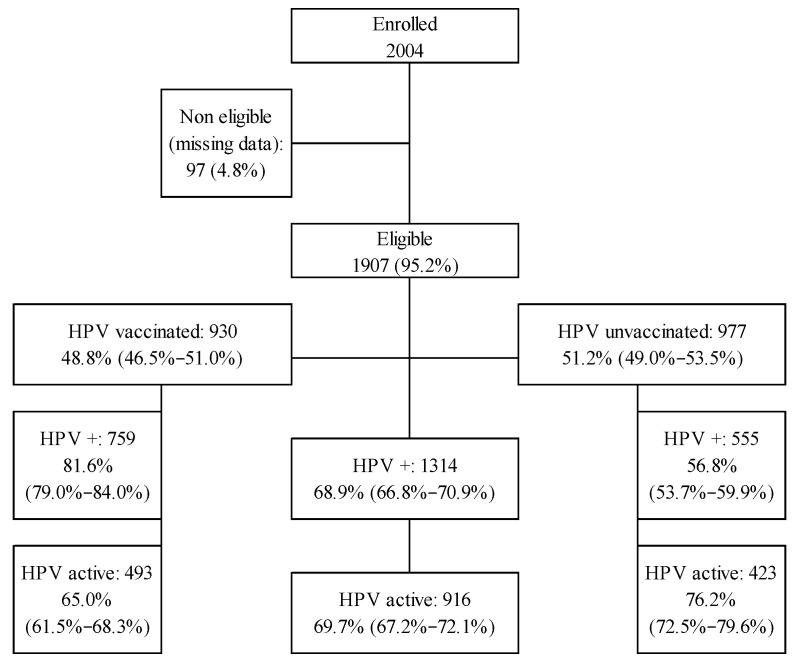
Participants’ flow chart in the study.

A total of 2004 women were enrolled in this cross-sectional study, of whom 97 (4.8%) were deemed ineligible for analysis due to missing data on vaccination status and/or HPV infection status. This study achieved geographical representativeness, with participants recruited across all regions of Spain, proportionally reflecting regional population densities according to the 2020 European Health Survey in Spain (see Supplementary Materials Tables S1–S4). Among participants, 72.6% were recruited from outpatient clinics within the public healthcare system and 27.4% from private hospitals. The mean age of the eligible women included in the analysis was 38.9 years (SD = 10.5), and 85.5% were of Spanish origin, closely mirroring the actual distribution of residents in Spain by nationality. Figure 1 presents the flowchart of the 1907 women considered eligible for the study. A total of 930 women (48.8% [95% CI: 46.5–51.0%]) reported antecedents of HPV vaccination, and 1314 (68.9% [66.8–70.9%]) reported antecedents of HPV infection. HPV infection was reported in 81.6% (79.0–84.0%) of vaccinated participants compared with 56.8% (53.7–59.9%) of unvaccinated participants (*p* < 0.001; Table 1). The mean age at vaccination was 31.0 years (SD = 11.9) (median: 31; interquartile range [IQR]: 23.0–38.3). The 9-valent vaccine was administered to 50.6% of vaccinated women (54.6% among HPV-positive women), while the remainder received either the 2-valent or 4-valent vaccines. Overall, 72.5% completed the three-dose schedule. Recurrent HPV infection after vaccination was observed in 33.8% (30.8–37.0%) of vaccinated participants, and 62.4% (56.5–67.9%) of these cases involved high-risk HPV types. Among women who developed abnormal cytology following vaccination, 46.9% (41.1–52.8%) presented with high-risk cytological abnormalities after vaccine administration. The 9-valent vaccine was statistically associated with a lower rate of recurrent HPV infection post-vaccination (Table 1): 27.3% (22.6–32.6%) compared with 35.2% (27.9–43.2%) and 40.8% (33.4–48.6%) for the 4- and 2-valent vaccines, respectively (*p* = 0.011). After adjustment for age at vaccination and number of doses received, recurrence rates remained significantly higher for the 4- and 2-valent vaccines compared with the 9-valent formulation (*p* = 0.009), with ORs of 1.45 (0.94–2.26; *p* = 0.096) and 1.94 (1.27–2.97; *p* = 0.002), respectively. Adverse events were reported by 2.8% of vaccinated women (26/930), all of which were mild in severity. The most frequently reported side effects were arm pain and fever (0.4% in both cases) (see Supplementary Materials Table S5).

Table 1 also summarizes the demographic and clinical characteristics of participants according to HPV vaccination status. Vaccinated women were more frequently of Spanish nationality, younger, had higher educational attainment, were less likely to be menopausal, and had fewer pregnancies and lower parity. Vaccinated women exhibited a higher prevalence of HPV infection compared with unvaccinated women and reported significantly greater overall use of contraception, including higher use of oral contraceptive pills. Regarding gynecological history, vaccinated women had significantly higher rates of cervical conization, genital warts, and abnormal cytology (with a lower frequency of ASC-US but higher rates of high-grade SIL and AGC; see Table 1 for acronyms), as well as a higher prevalence of cervical intraepithelial neoplasia of any grade (*p* < 0.001). HPV-related knowledge, assessed using the HPV-know-Q questionnaire, was significantly higher among vaccinated women compared with unvaccinated participants (Table 1, *p* < 0.001), although the magnitude of the difference was small (Cohen’s d = 0.38 [95% CI: 0.29–0.47]). This difference was attributable to significantly higher rates of correct responses among vaccinated women in up to 13 of the 20 individual questionnaire items (see Supplementary Materials Table S6).

### 3.1. Health Outcomes in HPV-Positive Women According to Vaccination Status

In unadjusted analyses, several clinical and health characteristics were significantly associated with HPV vaccination among HPV-positive women included in the study (Table 2). Vaccinated women were more frequently of Spanish nationality, younger, had a lower body mass index, and were less often menopausal compared with unvaccinated women. University education, nulligravidity, nulliparity, and use of any form of contraception were significantly more common among vaccinated women. Adequate information about HPV provided by a physician was numerically higher among vaccinated HPV-positive women compared with unvaccinated women (72.3% (69.0–75.4%) vs. 67.7% (63.8–71.5%), although this difference reached only borderline statistical significance (*p* = 0.089). Active HPV infection was significantly more frequent in unvaccinated women (Table 2): 79.7% (76.0–82.9%) compared with 65.9% (62.4–69.2%) in vaccinated participants (*p* < 0.001). Similarly, HPV infection within the previous 12 months was significantly more common among unvaccinated women (43.3% (39.2–47.4%) vs. 34.3% (31.0–37.7%); *p* = 0.007). Conversely, cervical conization, abnormal cytology, and CIN of any grade were significantly more frequent in vaccinated women. The mean age at vaccination was 33.3 years (SD = 10.8) (median: 33; IQR: 27.0–40.0). A total of 54.6% of women received the 9-valent vaccine, while the remainder received either the 2-valent or 4-valent vaccines; 76.2% completed the three-dose schedule. Recurrent HPV infection after vaccination was observed in 39.9% (36.4–43.5%) of vaccinated participants, with 62.4% (56.3–68.0%) attributable to high-risk HPV types. Among women who developed abnormal cytology after vaccination, 39.6% (36.0–43.2%) presented high-risk cytological abnormalities. The 9-valent vaccine was again associated with a significantly lower rate of recurrent HPV infection (Table 2): 29.5% (24.4–35.1%) compared with 41.3% (33.0–50.2%) and 47.9% (39.0–56.8%) for the 4-valent and 2-valent vaccines, respectively (*p* = 0.001). After adjustment for age at vaccination and number of doses received, recurrence remained significantly higher for the 4-valent and 2-valent vaccines compared with the 9-valent formulation (*p* = 0.002), with ORs of 1.64 (1.04–2.58; *p* = 0.032) and 2.21 (1.41–3.47; *p* = 0.001), respectively.

PROs are presented in Table 3. No statistically significant unadjusted differences were observed between vaccinated and unvaccinated HPV-positive women in total or the subdomain PRO scores assessed in this study. Similarly, no significant differences were found in the total or subscale scores of the HPV-QoL questionnaire between groups (Table 3). However, individual items Q1 (“Having HPV infection has changed my life”) and Q8 (“I feel worried about infecting my partner/those around me”) differed significantly in unadjusted comparisons, with higher Q1 scores among unvaccinated women and higher Q8 scores among vaccinated women (see Supplementary Materials Table S8). HPV knowledge, assessed in all participants using the HPV-Know-Q questionnaire, was significantly higher among vaccinated HPV-positive women compared with unvaccinated participants (Table 3, *p* = 0.001), although the effect size was negligible (Cohen’s d = 0.19 [0.08–0.31]). This difference was attributable to significantly higher frequencies of correct responses among vaccinated women in up to six individual questionnaire items (see Supplementary Materials Table S7).

### 3.2. Determinants of Health Outcomes According to HPV Vaccination Status

Multivariate logistic regression analysis in the overall sample demonstrated that, compared with unvaccinated women, the likelihood of HPV vaccination was significantly higher among women with HPV infection, Spanish nationality, use of any type of contraception, and, most notably, a history of cervical surgery (conization). In contrast, increasing age (years) and higher body mass index were negatively associated with the likelihood of HPV vaccination (Table 4). Cervical conization was the most strongly weighted determinant, with an OR for vaccination exceeding seven compared with women without prior conization (95% CI: 5.34–10.47; *p* < 0.001). Notably, greater HPV knowledge was also associated with a higher likelihood of vaccination; higher HPV-Know-Q scores corresponded to an increased probability of vaccination (OR = 1.02 [1.01–1.04]; *p* = 0.001).

**Figure 2:**
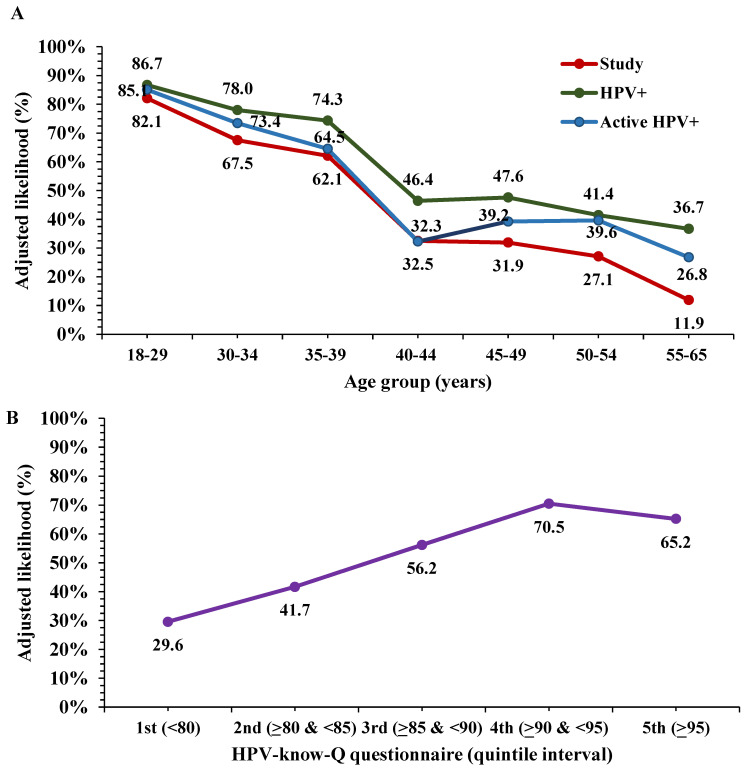
Adjusted likelihood (percentage) of HPV vaccination by age groups in the study population overall and according to HPV infection status (**A**), and by HPV knowledge based on the HPV−Know−Q questionnaire (**B**). Adjusted likelihood was estimated using determinants identified as significant in logistic regression models. Linear χ^2^: 314.25 (overall), 148.02 (HPV+), 129.83 (active HPV+), and 214.37 (HPV−Know−Q); *p* < 0.001 in all cases.

**Figure 3:**
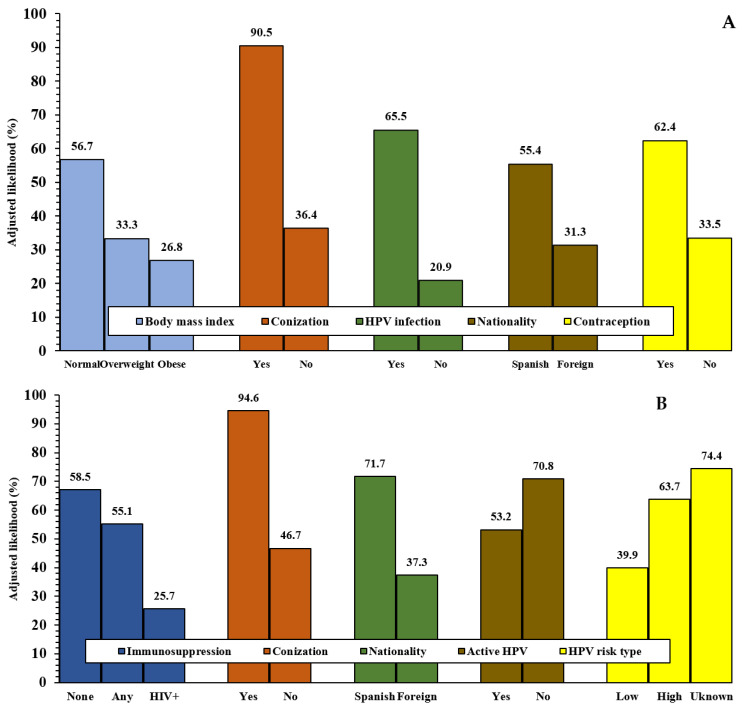
Adjusted likelihood (in percentage) of HPV vaccination in study determinants of vaccination in all study samples (**A**) and in HPV+ infected women (**B**). HPV = human papillomavirus; HIV+ = human immunodeficiency virus. Body mass index in kg/m^2^ (normal: <27; overweight: >27 and <30; obese: >30). Adjusted likelihood according to determinants identified as significant in logistic regression models. All group differences were statistically significant (*p* < 0.001).

Adjusted vaccination probability demonstrated a significant inverse linear association with increasing age categories (linear χ^2^: 314.25, *p* < 0.001) (Figure 2A), whereas it increased significantly with higher HPV knowledge scores (linear χ^2^: 214.37, *p* < 0.001) (Figure 2B). Adjusted probabilities of vaccination according to determinants significant at *p* < 0.001 are shown in Figure 3A. The probability of HPV vaccination exceeded 90% among adult women with a history of cervical surgery (90.5% vs. 36.4% in women without such a history; *p* < 0.001), representing the determinant with the greatest magnitude of association.

Table 4 also presents the clinical and health-related determinants in the HPV-positive subgroup. Spanish nationality, infection with high-risk or unknown HPV genotypes, and prior cervical conization were significantly associated with an increased likelihood of HPV vaccination. Conversely, increasing age, active HPV infection, and antecedents of HIV infection were negatively associated with vaccination likelihood. Cervical conization again emerged as the strongest explanatory determinant, with an OR nearly six times higher than in women without this history (95% CI: 4.22–8.28; *p* < 0.001). Adjusted vaccination probability demonstrated a significant inverse linear association with age categories (linear χ^2^: 148.02, *p* < 0.001) and with active HPV infection (linear χ^2^: 129.83, *p* < 0.001) (Figure 2A). Adjusted vaccination probabilities for determinants significant at *p* < 0.001 in HPV-positive women are presented in Figure 3B. Among infected participants with a history of cervical surgery, the likelihood of vaccination approached 100% (94.6% vs. 46.7% in women without prior surgery; *p* < 0.001). Another statistically significant determinant was the HPV genotype risk category, with a higher adjusted vaccination probability observed among women infected with high-risk HPV genotypes, particularly among those with an unknown genotype risk.

Although age upon first sexual intercourse encounter and number of sexual partners were excluded as independent determinants in multivariate models, adjusted vaccination probability demonstrated significant linear associations with both age at sexual debut and number of sexual partners (*p* < 0.001 for both; see Supplementary Materials Figures S1 and S2).

## 4. Discussion

To our knowledge, studies assessing determinants and factors related to HPV vaccination beyond HPV vaccine knowledge, acceptability, or attitudes toward vaccination in girls, adolescents, and their parents have been widely published in Spain and worldwide [19,20,21,22,23,24,34,35,36,37,38,39,40]. However, research examining health outcomes other than attitudes or acceptance as determinants related to HPV vaccination in adult women remains scarce [25,41,42]. In our study, 48.8% of adult women reported antecedents of HPV vaccination, which is slightly higher than initially estimated, although within the range reported in an attitudinal HPV vaccination study conducted in a similar population of adult women across several European countries [25]. As expected, vaccine uptake showed a significant inverse linear association with participant age: the older the age group, the lower the vaccination uptake. This pattern was observed in the overall sample, as well as in the HPV-positive and active HPV infection subsamples, particularly among women older than 40 years. The age limit established in the Spanish national vaccination program (45 years, except for women who have undergone conization, for whom there is no upper age limit for vaccination) may contribute to the mistaken perception that HPV vaccination should not be administered beyond that age. Although the European Union vaccine license does not currently include a maximum age limit for administration, this is also the case under US Food and Drug Administration (FDA) regulations [43,44,45,46].

Women’s knowledge about HPV, as assessed by the HPV-Know-Q questionnaire [28], was identified as a determinant of vaccine uptake. Higher knowledge scores were associated with a greater adjusted likelihood of vaccination, ranging from lower uptake rates among women scoring below 80% to approximately two-thirds among those scoring above 90%. More recent studies have also reported a positive correlation between increased HPV knowledge, greater perception of associated health risks, and higher vaccination acceptance [25,42,47]. The adjusted likelihood of HPV vaccination was significantly higher in HPV-positive women than in non-infected women (65.5% vs. 20.9%), which contrasts with the significantly higher likelihood of vaccination among women with past HPV infection compared with those with active infection (70.8% vs. 53.2%). This finding may be driven by increased motivation to protect oneself following awareness of infection, together with the documented positive influence of physician counseling and healthcare professional advice [25,34,36]; although in our study, the slightly higher proportion of vaccinated women reporting adequate physician-provided information showed only a trend toward statistical significance. This apparent inconsistency may be explained by gynecological antecedents (e.g., conization), which may encourage women to receive the HPV vaccine, as recently reported [48]. In our study, a history of conization was identified as the factor most strongly associated with HPV vaccination, with uptake 5–7 times higher among women with a history of cervical surgery compared with those without such antecedents, and an adjusted likelihood of vaccination of approximately 90%. However, this finding should not be interpreted as evidence that conization itself represents a behavioral determinant of vaccine uptake. Rather, conization should be considered a marker of prior HPV-related cervical disease and, in the Spanish context, a proxy for intensified interaction with the healthcare system, specialist follow-up, explicit clinical recommendation, and access to publicly funded vaccination after treatment. Therefore, the strong association observed between conization and vaccination probably reflects a healthcare-system-driven opportunity for vaccination rather than an intrinsic individual predisposition or behavioral pattern among women who underwent cervical surgery. This interpretation is consistent with the fact that vaccination in adult women may be more frequently offered or accepted after an HPV-related complication has occurred, particularly when the recommendation is delivered by gynecologists during post-treatment follow-up. Accordingly, these findings highlight the effectiveness of clinical pathways after conization, while also suggesting missed opportunities for proactive vaccination counseling in women without prior cervical disease. In the Spanish context, vaccination in adult women is often driven by the presence of existing lesions rather than as a proactive primary preventive measure. It may also indicate that the Spanish healthcare system is highly effective in recommending vaccination once a woman has experienced a HPV-related complication, but less effective in promoting vaccination among healthy or older women. In addition, the fact that the vaccine is provided free of charge in cases of conization may represent an additional motivating factor.

Another relevant determinant identified in this study was nationality. Multivariate models showed that foreign women residing in Spain had a significantly lower likelihood of having been vaccinated against HPV, both in the overall sample and in the HPV-positive subsample of adult women. The likelihood of HPV vaccination was approximately one-third among foreign residents, compared with more than 50% among Spanish women and more than 70% among infected Spanish participants. This observation is consistent with previous reports indicating that migrants globally face complex individual-, family, social-, provider-, and system-level barriers to HPV vaccination, resulting in low uptake and missed opportunities for protection [41], and it may pose a challenge for public health authorities, as these barriers limit equity in cancer prevention.

HPV-positive women showed a significantly higher likelihood of vaccination depending on the risk status of the infecting HPV serotype, with a higher likelihood when the serotype was high risk and, interestingly, when the risk status was unknown. The substantial protection against HPV16/18-related CIN2+ and the consequent shift in HPV genotype distribution among vaccinated women underscore the need to adapt screening strategies [49]. Contraception was another determinant associated with HPV vaccination in the overall sample: 62.4% of women using any type of contraception were expected to be vaccinated, compared with 33.5% among non-users. However, our findings contrast with recent studies in adolescents, university students, and young adult women attending cancer screening centers, which reported that HPV vaccination did not affect sexual behavior, attitudes toward condom use, or contraceptive choice [50,51]. Differences in age between study populations may explain this discrepancy.

Concomitant human immunodeficiency virus (HIV) infection among women with antecedents of HPV infection was associated with a lower likelihood of HPV vaccination compared with women receiving immunosuppressive therapy or none (25.7% vs. 55.1% and 58.5%, respectively). These findings are consistent with the work of Boivin et al. [52], who also reported high co-infection rates but lower HPV vaccination rates among HIV-positive women despite the substantial number of HIV-positive patients who could benefit from HPV vaccination.

Although no significant differences between vaccinated and unvaccinated adult women were found, moderate-to-severe anxiety symptoms (assessed using the HADS) were observed in one-third of the recruited women, clearly higher than the 9.2% prevalence of chronic anxiety reported among women of the same age range in the general Spanish population in the 2020 European Health Survey [53]. One possible explanation is that the study sample was recruited from gynecology and lower genital tract outpatient clinics, which may itself be associated with increased anxiety; however, only 14.7% of participants who completed the HADS were receiving antidepressant therapy, irrespective of vaccination status. Anxiety symptoms have also been documented in HPV-positive women attending cervical screening centers [5,7,8,25]. Another confirmatory finding was the high prevalence of sexual dysfunction (over 50% of participants), observed regardless of vaccination status or HPV infection, as assessed using the FSFI. All questionnaire domains contributed similarly to the total score, suggesting that all facets of female sexuality were affected to a comparable extent. Previous studies indicate that HPV-positive women experience significantly higher rates of sexual dysfunction, with lower scores across most domains (arousal, lubrication, orgasm, satisfaction, and pain). High-risk strains such as HPV16/18 have been associated with higher anxiety and greater reductions in sexual desire and lubrication [4,7,51,54]. HRQoL, assessed using the HPV-specific instrument (HPV-QoL scale), showed scores around the median value of Spanish normative data [55], both in vaccinated and unvaccinated women, irrespective of HPV infection status. We cannot directly compare the quality of life of women in this study with that reported in studies describing poor quality of life in HPV-infected women [6,7,56], as the objective of this study was to compare vaccinated versus unvaccinated women. In the unadjusted analysis, questions Q1 (“Having HPV infection has changed my life”) and Q8 (“I feel worried about infecting my partner/those around me”) of the HPV-QoL questionnaire differed significantly between vaccinated and unvaccinated women, with higher scores for Q1 among unvaccinated women and for Q8 among vaccinated women (see Supplementary Materials). These findings are difficult to interpret but may suggest that women who strongly disagree that HPV infection has changed their life are more likely to decline vaccination, whereas those who strongly agree that they feel worried about infecting their partners or others may be more likely to be vaccinated. Mental distress, assessed using the GHQ-12, showed no differences between vaccinated and unvaccinated women beyond the well-established correlation between mental distress and HRQoL in HPV-infected women, whereby higher mental distress corresponds to lower quality of life [32,55]. However, the prevalence of mental distress (approximately one-fourth of participants) is consistent with published studies reporting high levels of anxiety and/or depression among women attending cervical cancer screening centers [4,7,25,56].

The type of data analyzed regarding HPV vaccination in adult women aged 18–65 years, the large sample size (more than 1900 adult women), and its interterritorial, nationwide scope, which provides a certain degree of representativeness, contribute to the robustness and relevance of the statistical analysis for researchers in the fields of both HPV vaccination and HPV infection. In addition, the use of validated multidimensional instruments and the focus on a population that has been scarcely studied (adult Spanish women in a real-world setting) enhance the novelty of this study.

However, several limitations should be acknowledged. First, only women attending gynecology and lower genital tract outpatient clinics were included, which may limit the generalizability of the findings to the broader population. This is because the observed prevalence of HPV infection and vaccination uptake could be biased, favoring higher figures than in the general population, as a consequence of enrolling participants at higher risk of both HPV infection and vaccination uptake. Men were not included despite also being susceptible to HPV infection and included in vaccination recommendations. The completion rate of the HADS was low (45% of HPV-positive participants), precluding its inclusion as an explanatory variable in multivariate analyses. Other PROM instruments were completed by at least 85% of participants. Finally, due to its cross-sectional design, findings in this study must be considered associative rather than causal. Also, the stepwise regression models applied, even when using a large sample, might have limitations, including, amongst others, bias in parameter estimation or misinterpretation of *p*-values, making them misleadingly low. Because of this limitation, the temporal and causal relationship between conization and HPV vaccination cannot be established. The association may reflect confounding by indication, physician recommendation, reimbursement policies, and greater healthcare contact after treatment rather than a behavioral effect attributable to the patient.

## 5. Conclusions

In conclusion, this nationwide study of adult women aged 18–65 years attending gynecology and lower genital tract outpatient clinics, which is the population to whom the findings are applicable, found a HPV vaccination uptake rate of 48.8%, which is acceptable but clearly warrants improvement, irrespective of HPV infection status. Uptake was 81.6% among women who were HPV positive but was only 65.9% among those with an active infection, which acted as a negative factor for vaccination. Thus, the possibility of reverse causation (HPV diagnosis prompting vaccination) cannot be ruled out. Increasing age was another negative factor, with a decreasing likelihood of vaccination as age increased, particularly after 40 years. Improvement in uptake could be achieved by extending vaccination recommendations up to 65 years of age, given that no age limits currently exist for HPV vaccine use from a regulatory perspective, and by increasing the involvement of healthcare professionals in vaccination counseling and support. Simply informing patients is insufficient; active clinical recommendations and the removal of perceived age limits (beyond 45 years of age) by physicians are required to improve coverage rates. In our study, women’s knowledge about HPV and adequate physician-provided information were associated with a higher likelihood of vaccination. This study also identified additional health outcome determinants favoring vaccination beyond HPV infection, such as a higher uptake among women with a history of cervical surgery, contraceptive use, or infection with high-risk or unknown-risk HPV serotypes, highlighting the role of gynecologists in promoting vaccination. Finally, the likelihood of vaccination was substantially higher among Spanish women compared with foreign residents in Spain, representing a challenge for public health authorities and the medical community, as migrants should also be encouraged to participate in HPV preventive programs without constraints. HPV vaccination campaigns focusing on HPV, irrespective of age, sex, or origin, should be endorsed by public health authorities to reach the objective of virus eradication.

## Figures and Tables

**Table 1:** Demographic and clinical characteristics of 1907 women by HPV vaccination status in the study.

Characteristic	Total (n = 1907)	HPV Vaccination		
		No (n = 977, 51.2%)	Yes (n = 930, 48.8%)	*p*
Nationality (Spanish), %	85.5%	82.7%	88.4%	<0.001
Age (years), mean (SD)	38.9 (10.5)	41.7 (10.6)	36.1 (9.6)	<0.001
BMI (kg/m^2^), mean (SD)	24.3 (10.5)	25.3 (12.9)	23.3 (7.2)	<0.001
Educational background, %				
Primary or lower secondary	8.4	10.2	6.5	0.002
Upper secondary/vocational training	30.3	31.6	28.9	
University	61.3	58.2	64.6	
Menopause, %	15.2	20.3	10.0	<0.001
Sexually active				
No	16.3	17.3	15.3	0.251
Occasional	21.1	21.9	20.3	
Regular	62.5	60.7	64.4	
Partner, %				
No	3.5	3.2	3.8	0.716
Occasional	27.6	27.3	27.9	
Stable	69.0	69.6	68.3	
Type of sexual partner, %				
Man	94.6	95.2	94.0	0.482
Woman	2.8	2.6	3.0	
Both	2.6	2.2	3.0	
Age at first sexual intercourse, mean (SD)	18.1 (3.3)	18.5 (3.3)	17.8 (2.9)	<0.001
Number of partners,	8.8 (12.8)	8.5 (14.2)	9.2 (11.0)	0.273
Gestations, %				
0	48.5	37.6	60.0	<0.001
1	19.8	21.4	18.0	
2	18.9	24.8	12.7	
3+	12.8	16.2	9.3	
Deliveries, %				
0	55.6	45.2	66.4	<0.001
1	20.8	24.5	17.0	
2	18.1	23.5	12.5	
3+	5.5	6.8	4.1	
Immunosuppression, %				
No	93.7	92.4	95.1	0.017
Yes (no HIV)	3.7	4.0	3.3	
HIV	2.6	3.6	1.6	
Smoking (1 or more cigarettes per day), %	23.1	22.2	24.1	0.221
Alcohol (daily consumption), %	2.1	2.1	2.1	0.881
Exercise, %				
No	19.2	20.2	18.2	0.295
Sometimes	39.1	39.8	38.5	
Regular	41.7	40.1	43.4	
Contraception, %	64.5	56.4	72.9	<0.001
Type of Contraception, %				
Condom	63.2	62.5	63.8	0.001
Píll	20.6	17.0	23.5	
Implant	1.8	2.8	1.1	
Hormonal IUD	5.7	6.7	5.0	
Copper IUD	4.3	4.8	3.9	
Tubal ligation/vasectomy	4.4	6.3	2.9	
Previous STI, %	14.4	13.8	15.0	0.480
Cervical conization, %	28.7	13.7	44.1	<0.001
Genital warts, %	15.3	13.3	17.4	0.017
Abnormal cytology, %	55.3	46.0	65.0	<0.001
Type				
ASC-US	50.8	55.9	47.1	0.007
ASC, High grade	16.3	16.4	16.3	
Low-grade SIL	1.7	2.2	1.4	
High-grade SIL	15.4	13.7	16.6	
AGC	12.1	8.1	15.0	
CANCER	0.3	0.0	0.5	
Unknown	3.3	3.7	3.0	
CIN				
No	27.4	38.0	19.7	<0.001
Grade I	10.1	8.5	11.3	
Grade II	13.0	5.8	18.1	
Grade III	11.5	7.1	14.8	
Unknown	38.0	40.6	36.1	
HPV infection (any time), %	68.9	56.8	81.6	<0.001
HPV-Know-Q questionnaire (woman’s knowledge on HPV), mean (95% CI)	86.3 (85.8–86.8)	84.3 (83.5–85.0)	88.3 (87.7–89.0)	<0.001
Vaccination data				
Age (years) at vaccination, mean (SD)	-	-	31.0 (11.9)	-
Type of HPV vaccine, %			620/930 (66.7)	
2-valent	-	-	25.8	-
4-valent	-	-	23.5	-
9-valent	-	-	50.6	-
Number of doses administered, %			906/930 (97.4)	
One	-	-	7.3	-
Two	-	-	20.2	-
Three	-	-	72.5	-
HPV after vaccination, n (%)	-	-	301/891 (33.8)	
Type of HPV virus by risk after vaccination, %				
Low	-	-	102/271 (37.6)	
High	-	-	169/271 (62.4)	-
HPV after vaccination by type of vaccine				
2-valent	-	-	64/157 (40.8)	0.011
4-valent	-	-	51/145 (35.2)	
9-valent	-	-	82/300 (27.3)	
Abnormal cytology after vaccine, n %	-	-	302/867 (34.8)	-
High-risk cytology, n %	-	-	129/275 (46.9)	-

Values are unadjusted means or percentages; HPV = human papilloma virus; SD = standard deviation; BMI = body mass index; HIV = human immunodeficiency virus; STI = sexually transmitted infection; IUD = Intrautereine Device; ASC-US = atypical squamous cells of undetermined significance; CIN = cervical intraepithelial neoplasia; SIL = Squamous Intraepithelial Lesion (SIL)**;** AGC = atypical glandular cells lesion.

**Table 2:** Demographic and clinical characteristics of women with HPV+ infection by HPV vaccination status in the study.

Characteristic	HPV+ (n = 1314)	HPV Vaccination		
		No (n = 555, 42.2%)	Yes (n = 759, 57.8%)	*p*
Nationality (Spanish), %	83.1%	78.4%	86.6%	<0.001
Age (years), mean (SD)	38.8 (9.8)	40.8 (10.4)	37.4 (9.1)	<0.001
BMI (kg/m^2^), mean (SD)	24.0 (9.1)	25.2 (13.1)	23.2 (4.3)	0.001
Educational background, %				
Primary or lower secondary	7.6	8.4	7.0	0.007
Upper secondary/vocational training	31.8	36.1	28.7	
University	60.6	55.5	64.2	
Menopause, %	13.7	17.3	11.1	<0.001
Sexually active				
No	16.8	18.4	15.6	0.070
Occasional	21.8	23.9	20.4	
Regular	61.4	57.7	64.0	
Partner, %				
No	2.5	2.2	2.8	0.148
Occasional	31.4	34.2	29.3	
Stable	66.1	63.6	67.9	
Type of sexual partner, %				
Man	94.4	94.9	94.1	0.816
Woman	2.8	2.6	3.0	
Both	2.8	2.6	3.0	
Number of partners,	10.5 (13.9)	11.1 (16.4)	10.1 (11.8)	0.233
Age at first sexual intercourse, mean (SD)	18.0 (3.0)	18.1 (3.2)	17.9 (2.9)	0.118
Gestations, %				
0	51.7	44.3	57.1	<0.001
1	20.2	21.4	19.3	
2	16.7	21.0	13.6	
3+	11.4	13.3	10.0	
Deliveries, %				
0	58.6	52.2	63.2	0.006
1	21.1	24.6	18.5	
2	16.0	19.0	13.8	
3+	4.3	4.2	4.5	
Immunosuppression, %				
No	92.8	90.7	94.4	0.010
Yes (no HIV)	4.0	4.4	3.7	
HIV	3.1	4.8	1.9	
Smoking (1 or more cigarettes per day), %	26.0	27.3	25.1	0.354
Alcohol (daily consumption), %	2.5	2.9	2.3	0.393
Exercise, %				
No	19.3	19.1	19.6	0.453
Sometimes	39.3	38.1	40.9	
Regular	41.4	42.8	39.5	
Contraception, %	68.6	63.2	72.6	<0.001
Type of Contraception, %				
Condom	66.0	67.0	65.4	0.086
Píll	20.4	18.0	21.9	
Implant	1.7	2.6	1.1	
Hormonal IUD	4.4	3.8	4.8	
Copper IUD	3.5	2.9	3.9	
Tubal ligation/vasectomy	4.1	5.8	2.9	
Previous STI, %	18.1	19.4	17.1	0.320
Cervical conization, %	39.2	20.7	52.4	<0.001
Genital warts, %	20.2	20.0	20.3	0.956
Abnormal cytology, %	70.4	64.7	74.5	<0.001
Type				
ASC-US	49.5	52.6	47.6	0.151
ASC, High grade	16.4	17.1	16.0	
Low-grade SIL	1.6	2.1	1.3	
High-grade SIL	16.2	15.9	16.4	
AGC	13.2	9.3	15.6	
CANCER	0.2	0.0	0.4	
Unknown	2.9	3.0	2.8	
CIN				
No	23.7	34.3	17.0	<0.001
Grade I	10.7	9.0	11.8	
Grade II	14.5	7.2	19.1	
Grade III	12.7	7.8	15.7	
Unknown	38.4	41.8	36.3	
Active HPV infection, %	71.6	79.7	65.9	<0.001
Elapsed time in case of HPV active infection (months), mean (SD)	34.4 (105.2)	31.9 (112.2)	36.4 (99.3)	0.549
Active infection in last 12 months, %	38.4	43.3	34.3	0.007
Type of HPV virus by risk, %				<0.001
Low	19.4	25.1	15.2	
High	24.5	26.5	23.1	
Unknown	56.1	48.4	61.7	
Information on HPV given by her doctor (Grade: adequate), %	70.4	67.7	72.3	0.089
Vaccination data				
Age (years) at vaccination, mean (SD)	-	-	33.3 (10.8)	-
Type of HPV vaccine, %			533/759 (70.2)	
2-valent	-	-	22.5	-
4-valent	-	-	22.9	-
9-valent	-	-	54.6	-
Number of doses administered, %			748/759 (98.6)	
One	-	-	6.3	-
Two	-	-	17.5	-
Three	-	-	76.2	-
HPV after vaccination, n (%)	-	-	289/724 (39.9)	
Type of HPV virus by risk after vaccination, %				
Low	-	-	98/260 (37.7)	
High	-	-	162/260 (62.3)	-
HPV after vaccination by type of vaccine				
2-valent	-	-	56/117 (47.9)	0.001
4-valent	-	-	50/121 (41.3)	
9-valent	-	-	82/278 (29.5)	
Abnormal cytology after vaccine, n %	-	-	279/705 (39.6)	-
High risk cytology, n %	-	-	122/254 (48.0)	-

Values are unadjusted means or percentages; HPV = human papilloma virus; SD = standard deviation; BMI = body mass index; HIV = human immunodeficiency virus; STI = sexually transmitted infection; IUD = Intrautereine Device; ASC-US = atypical squamous cells of undetermined significance; CIN = cervical intraepithelial neoplasia; SIL = Squamous Intraepithelial Lesion; AGC = atypical glandular cells lesion.

**Table 3:** Patient-reported outcomes (PROs) of women with HPV+ infection by HPV vaccination status in this study.

Characteristic	HPV+ (n = 1314, 68.9%)	HPV Vaccination		
		No (n = 555, 56.8%)	Yes (n = 759, 81.6%)	*p*
HAD scale (n = 591)				
Anxiety domain	9.0 (8.6–9.4)	8.9 (8.3–9.4)	9.1 (8.6–9.6)	0.525
Anxiety (Moderate/Severe; ≥11 points), n (%)	196/591 (33.2)	74/249 (29.7)	122/342 (35.7)	0.646
Depression domain	4.7 (4.4–5.1)	4.8 (4.3–5.3)	4.6 (4.2–5.1)	0.498
Depression (Moderate/Severe; ≥11 points), n (%)	59/591 (10.0)	25/249 (10.0)	34/342 (10.0)	0.969
FSFI questionnaire (n = 1104)				
Total score	21.7 (21.0–22.3)	21.4 (20.4–22.5)	21.8 (21.0–22.7)	0.540
Sexual dysfunction (score ≤ 26.55 points), n (%)	577/1104 (52.3)	236/443 (53.3)	341/661 (51.6)	0.583
Desire	3.4 (3.3–3.5)	3.3 (3.2–3.5)	3.4 (3.3–3.5)	0.268
Arousal	3.5 (3.4–3.6)	3.5 (3.3–3.7)	3.6 (3.4–3.7)	0.468
Lubrication	3.7 (3.6–3.8)	3.6 (3.4–3.8)	3.7 (3.6–3.9)	0.447
Orgasm	3.7 (3.6–3.8)	3.7 (3.5–3.9)	3.7 (3.5–3.8)	0.965
Satisfaction	3.7 (3.6–3.9)	3.7 (3.5–3.9)	3.8 (3.6–3.9)	0.678
Pain	3.6 (3.5–3.8)	3.6 (3.4–3.8)	3.7 (3.5–3.9)	0.577
GHQ-12 questionnaire (n = 1185)				
Total composite score	13.5 (13.2–13.9)	13.5 (13.0–14.1)	13.5 (13.1–14.0)	0.999
Mental distress (score ≥ 17 points), n (%)	300/1185 (25.3)	127/480 (26.5)	173/705 (24.5)	0.456
Self-esteem	4.8 (4.6–5.0)	4.8 (4.5–5.1)	4.8 (4.5–5.0)	0.942
Stress	4.0 (3.9–4.1)	3.9 (3.8–4.1)	4.0 (3.9–4.1)	0.622
Coping	4.8 (4.7–4.9)	4.8 (4.6–4.9)	4.8 (4.6–4.9)	0.765
HPV-Know-Q questionnaire (person’s knowledge on HPV) (n = 1279)	88.4 (87.9–88.9)	87.3 (86.4–88.2)	89.2 (88.5–89.8)	0.001
HPV-QoL questionnaire (n = 1225)				
Total composite score	44.3 (43.2–45.5)	44.3 (42.5–46.1)	44.4 (42.9–45.8)	0.943
General wellbeing	53.3 (51.9–54.7)	53.9 (51.8–56.1)	52.9 (51.0–54.7)	0.467
Psychological wellbeing	42.0 (40.5–43.6)	42.8 (40.4–45.2)	41.5 (39.6–43.5)	0.427
Social wellbeing	75.8 (74.2–77.3)	76.1 (73.8–78.4)	75.5 (73.5–77.6)	0.693
Contagiouness	46.9 (45.5–48.2)	46.4 (44.2–48.5)	47.2 (45.5–48.9)	0.545
Health	20.7 (19.5–21.9)	21.3 (19.4–23.1)	20.3 (18.8–21.8)	0.444
Sexuality	56.5 (54.8–58.3)	55.7 (53.0–58.4)	57.1 (54.9–59.4)	0.412

Values are unadjusted means with 95% confidence intervals in parenthesis; HPV = human papilloma virus; HAD scale = hospital anxiety and depression scale (range 0–21 points in each of the two domains); FSFI = Female Sexual Function Index (full-scale score range is from 2 to 36); GHQ-12 = global health questionnaire-12 items (range 0–36); HPV-Know-Q questionnaire range: 0–100 full knowledge; HPV-QoL = HPV-Quality-of-life questionnaire, range 0 (worst QoL) to 100 (best QoL).

**Table 4:** Sociodemographic, clinical, and health outcome determinants of HPV vaccination.

All Study Sample	B Coefficient	SE	Wald	OR	95% CI		*p*
Determinant					Lower	Upper	
Age (years)	−0.09	0.01	127.77	0.92	0.90	0.93	<0.001
HPV infection	0.77	0.16	24.39	2.16	1.59	2.93	<0.001
Spanish nationality	1.03	0.20	25.54	2.79	1.87	4.15	<0.001
Contraception	0.40	0.14	8.08	1.49	1.13	1.96	0.004
Antecedents of HPV surgery (conization)	2.01	0.17	137.57	7.48	5.34	10.47	<0.001
Body mass index (kg/m^2^)	−0.03	0.01	5.21	0.97	0.95	1.00	0.022
Knowledge of HPV (HPV-Know-Q total score)	0.02	0.01	11.14	1.02	1.01	1.04	0.001
Constant	−0.15	0.71	0.04	0.86			0.838
**Women with HPV+ infection**	**B** **coefficient**	**SE**	**Wald**	**OR**	**95% CI**		***p***
**Determinant**					**Lower**	**Upper**	
Age (years)	−0.06	0.01	60.88	0.94	0.92	0.95	<0.001
Nationality (Spanish)	0.90	0.20	21.24	2.46	1.68	3.61	<0.001
Active HPV infection	−0.47	0.17	7.97	0.63	0.45	0.87	0.005
Immunosuppression (HIV+ infection)	−1.22	0.47	6.81	0.30	0.12	0.74	0.009
Antecedents of HPV surgery (conization)	1.78	0.17	106.81	5.91	4.22	8.28	<0.001
Infection with high-risk HPV type	0.62	0.21	8.60	1.86	1.23	2.82	0.003
Infection with unknown-risk HPV type	0.53	0.19	7.70	1.68	1.17	2.42	0.006
Constant	1.44	0.36	15.59	4.21			<0.001

HPV = human papillomavirus; HIV+ = human immunodeficiency virus; SE = standard error; OR = odds ratio vaccination; CI = confidence interval; HPV-Know-Q = Questionnaire on HPV knowledge (Q = Question: 0 = False, 1 = True); HPV-QoL = HPV-Quality-of-life questionnaire (Q = Question, range: from 1 = Totally agree to 5 = Totally disagree).

## Data Availability

The data presented in this study are openly available in https://redcap.idissc.org/redcap/surveys/?s=9HJDJKDTEP (accessed on 16 April 2026).

## Supplementary Materials

The following supporting information can be downloaded at: https://www.mdpi.com/article/10.3390/vaccines14050460/s1, Figure S1: Adjusted likelihood (percentage) of HPV vaccination according to age of women at first sexual relationship encounter, globally and by HPV infection status; Figure S2. Adjusted likelihood (percentage) of HPV vaccination according to number of sexual partners of participants’ women, globally and by HPV infection status; Table S1: Representativeness of sample used in the study against Spanish women general population in year 2020, as per the European Health Survey in Spain (EHSS 2020): regional distribution; Table S2: Representativeness of sample used in this study against Spanish women general population in year 2020, as per the European Health Survey in Spain (EHSS 2020): Educational level; Table S3: Representativeness of sample used in this study against Spanish women general population in year 2020, as per the European Health Survey in Spain (EHSS 2020): Age group; Table S4: Representativeness of sample used in this study against Spanish women general population in year 2020, as per the European Health Survey in Spain (EHSS 2020): Habits; Table S5: List of side effects reported in women receiving a HPV vaccine; Table S6: HPV-know-Q questionnaire unadjusted frequency of corrected answers, in percentage, by HPV vaccination status; Table S7: HPV-know-Q questionnaire unadjusted frequency of corrected answers, in percentage, by HPV vaccination status in the subsample of women with HPV+ infection; Table S8: HPV-QoL questionnaire unadjusted scores by individual questions according to HPV vaccination status in the subsample of 1314 women with HPV infection (Vaccination = 759; No vaccination = 555); Table S9: Missing data for each variable included in the study.

## Abbreviations

AGC	Atypical Glandular Cells
ANOVA	Analysis of Variance
ASC-US	Atypical Squamous Cells of Undetermined Significance
BMI	Body Mass Index
CI	Confidence Interval
CIN	Cervical Intraepithelial Neoplasia
EHSS	European Health Survey in Spain
FSFI	Female Sexual Function Index
GHQ-12	Goldberg Global Health Questionnaire
HAD scale	Hospital Anxiety and Depression Scale
HPV-QoL	HPV-Quality-of-life Questionnaire
HIV	Human Immunodeficiency Virus
HPV	Human Papillomavirus
IUD	Intrauterine Device
IQR	Interquartile Range
OR	Odds Ratio
PRO	Patient-Reported Outcomes.
SD	Standard Deviation
SIL	Squamous Intraepithelial Lesion
STI	Sexually Transmitted Infection
VCR	Vaccination Coverage Rate
WHO	World Health Organization
