# Supplementary material for: Health Outcome Determinants of Human Papillomavirus Vaccination in Adult Women in Spain
Source: Vaccines (Basel). 2026 May 21;14(5):460. doi: 10.3390/vaccines14050460 (PMC13211339; doi:10.3390/vaccines14050460)
Supplement: Supplementary file 1 [file vaccines-14-00460-s001.zip › vaccines-4248328-supplementary.pdf]

Supplementary Material

Supplementary Table S1. Representativeness of sample used in the study against Spanish women general population in year 2020, as per the European Health Survey in Spain (EHSS 2020): regional distribution.

Women 25- to 65-year-old distribution by geographical region (%)		
	EHSS 2020	This study
Andalucía	13.8	17.2
Aragón	3.4	11.9
Asturias, Principado de	4.4	0.5
Balears, Illes	2.0	0.2
Canarias	5.7	0.2
Cantabria	4.2	0.3
Castilla y León	4.0	4.1
Castilla - La Mancha	4.9	4.7
Cataluña	9.6	26.7
Comunitat Valenciana	8.2	1.5
Extremadura	4.0	0.5
Galicia	5.4	0.6
Madrid, Comunidad de	11.1	27.2
Murcia, Región de	4.5	3.3
Navarra, Comunidad Foral de	3,5	0.2
País Vasco	5.3	0.5
Rioja, La	2.9	0.2
Ceuta	1.3	0.2
Melilla	1.7	0.1
Unknown	-	
	100.0	100.0

Source: Ministerio de Sanidad y Consumo. Encuesta Europea de Salud en España 2020. Available at: https://www.mscbs.gob.es/estadEstudios/estadisticas/EncuestaEuropea/Enc_Eur_Salud_en_Esp_2020.htm. (Accessed on 28 July 2025).

Supplementary Table S2. Representativeness of sample used in this study against Spanish women general population in year 2020, as per the European Health Survey in Spain (EHSS 2020): Educational level.

Women 25 to 65 year old distribution by educational level (%)		
	EHSS 2020	This study
Primary	14.3	8.4
High school/LT	37.0	30.3
University/LT high degree trainee	48.7	61.3
	100.0	100.0

Source: Ministerio de Sanidad y Consumo. Encuesta Europea de Salud en España 2020. Available at: https://www.mscbs.gob.es/estadEstudios/estadisticas/EncuestaEuropea/Enc_Eur_Salud_en_Esp_2020.htm. (Accessed on 28 July 2025).

Supplementary Table S3. Representativeness of sample used in this study against Spanish women general population in year 2020, as per the European Health Survey in Spain (EHSS 2020): Age group.

Women 25- to 65-year-old distribution by age group (%)		
Age group (years)	EHSS 2020	This study
Mean (SD) age (years),	47.5 (10.9)	38.9 (10.5)
25-29	6.2	19.4
30-34	8.1	18.9
35-39	11.4	17.8
40-44	14.5	14.7
45-49	14.5	12.7
50-54	13.3	7.3
55+	32.0	9.0
	100.0	100.0

Source: Ministerio de Sanidad y Consumo. Encuesta Europea de Salud en España 2020. Available at: https://www.mscbs.gob.es/estadEstudios/estadisticas/EncuestaEuropea/Enc_Eur_Salud_en_Esp_2020.htm. (Accessed on 28 July 2025).

Supplementary Table S4. Representativeness of sample used in this study against Spanish women general population in year 2020, as per the European Health Survey in Spain (EHSS 2020): Habits.

Women 25- to 65-year-old distribution by habits (%)		
Age group (years)	EHSS 2020	This study
Daily smoking	22.1	23.1
Daily alcohol consumption	6.3	2.1
	100.0	100.0

Source: Ministerio de Sanidad y Consumo. Encuesta Europea de Salud en España 2020. Available at: https://www.mscbs.gob.es/estadEstudios/estadisticas/EncuestaEuropea/Enc_Eur_Salud_en_Esp_2020.htm. (Accessed on 28 July 2025).

Supplementary Table S5. List of side effects reported in women receiving a HPV vaccine.

Side effect	Frequency (#women)
local inflammation	1
dizziness and fever	1
vomiting, malaise	1
lump in arm	1
arm pain	4
muscle pain and dizziness	1
fever	4
lymph node swelling	1
general malaise	2
dizziness	1
dizziness, pain, nausea	1
rash	1
menstrual disorders	2
unknown	5

Supplementary Table S6. HPV-know-Q questionnaire unadjusted frequency of corrected answers, in percentage, by HPV vaccination status.

Question (item)	Total	HPV Vaccination		p
	(n=1,841)	No (n=929)	Yes (n=912)	
P 1. HPV is a virus: respiratory virus/sexual transmission /Like AIDS.	98.7	98.5	98.9	0.568
P 2. Are all HPV the same? Yes/ No, those with the highest number are the most dangerous/No, there are high and low risk.	85.8	83.4	88.2	0.005
P 3. HPV produces cervix cancer/discomfort in the vagina and vulva/It doesn´t affect to the human.	96.3	95.2	97.4	0.017
P 4. Which of the following cancers is HPV not related to? Vulva/anus/ lungs.	91.9	91.5	92.2	0.632
P 5. HPV affect to only women/only men/both.	85.1	82.0	88.3	<0.001
P 6. HPV vaccine: it is very useful/ it is useful only in girls/it produces serious adverse effects.	84.6	79.7	89.7	<0.001
P 7. Genital warts: only affect to the woman/they don´t have treatment/They are produced by HPV.	92.4	90.1	94.8	<0.001
P 8. VPH infection: the disease isn´t cured/Most women eliminate it over time/it can only be eliminated with vaccines.	75.9	71.0	80.9	<0.001
P 9. If I have HPV infection, the normal thing is that I have cancer/vulvar discomfort and itching/ nothing serious.	69.1	64.9	73.4	<0.001
P 10. Cytology (Papanicolaou test) uses to detect early cervix cancer/ fungal infection/ menstrual disorders.	96.1	95.5	96.8	0.170
P 11. If I have a stable partner, I can´t have HPV Infection.	94.0	92.2	95.8	0.002
P 12. If I Have HPV infection, my partner has been unfaithful.	88.8	86.2	91.4	<0.001
P 13. HPV infection is transmitted by sexual relations.	97.2	96.2	98.2	0.013
P 14. If I have HPV infection, I will have cervix cancer.	87.6	84.8	90.5	<0.001
P 15. If I get HPV infection, I can eliminate it. It won´t last forever.	79.6	78.1	81.0	0.140
P 16. If I always use a condom, I can´t get infected.	60.9	56.1	65.8	<0.001
P 17. HPV infection affects men and women equally.	53.9	56.0	51.9	0.085
P 18. If I am vaccinated against HPV, I don´t need to have cytology.	99.0	98.5	99.5	0.071
P 19. HPV infection can be purchased a long time ago.	94.7	93.8	95.7	0.074
P 20. Since I have an infection, I cannot get pregnant.	93.9	91.5	96.3	<0.001

p values were unadjusted by covariates. HPV=Human papillomavirus.

Supplementary Table S7. HPV-know-Q questionnaire unadjusted frequency of corrected answers, in percentage, by HPV vaccination status in the subsample of women with HPV+ infection.

Question (item)	Total (n=1,314)	No (n=555)	Yes (n=759)	p
P 1. HPV is a virus: respiratory virus/sexual transmission /Like AIDS.	99.1%	99.2%	99.1%	1.000
P 2. Are all HPV the same? Yes/ No, those with the highest number are the most dangerous/No, there are high and low risk.	88.6%	87.4%	89.5%	0.284
P 3. HPV produces Cervix cancer/discomfort in the vagina and vulva/It doesn´t affect to the human.	97.9%	97.2%	98.4%	0.191
P 4. Which of the following cancers is HPV not related to? Vulva/anus/ lungs.	92.4%	92.1%	92.7%	0.780
P 5. HPV affect to only women/only men/both.	89.4%	88.1%	90.3%	0.258
P 6. HPV vaccine: it is very useful/ it is useful only in girls/it produces serious adverse effects.	84.8%	78.1%	89.6%	<0.001
P 7. Genital warts: only affect to the woman/they don´t have treatment/They are produced by HPV.	94.6%	93.8%	95.2%	0.326
P 8. VPH infection: the disease isn´t cured/Most women eliminate it over time/it can only be eliminated with vaccines.	84.4%	85.7%	83.6%	0.350
P 9. If I have HPV infection, the normal thing is that I have cancer/vulvar discomfort and itching/ nothing serious.	76.4%	75.8%	76.8%	0.753
P 10. Cytology (Papanicolaou test) uses to detect early cervix cancer/ fungal infection/ menstrual disorders.	97.1%	96.2%	97.7%	0.158
P 11. If I have a stable partner, I can´t have HPV Infection.	94.3%	92.1%	95.9%	0.006
P 12. If I Have HPV infection, my partner has been unfaithful.	89.6%	86.8%	91.6%	0.007
P 13. HPV infection is transmitted by sexual relations.	98.4%	97.7%	98.8%	0.211
P 14. If I have HPV infection, I will have cervix cancer.	89.0%	86.8%	90.5%	0.045
P 15. If I get HPV infection, I can eliminate it. It won´t last forever.	82.3%	83.6%	81.4%	0.360
P 16. If I always use a condom, I can´t get infected.	63.4%	60.0%	65.8%	0.038
P 17. HPV infection affects men and women equally.	55.2%	57.7%	53.4%	0.140
P 18. If I am vaccinated against HPV, I don´t need to have cytology tests.	99.3%	98.7%	99.7%	0.060
P 19. HPV infection can be purchased a long time ago.	96.1%	95.3%	96.7%	0.268
P 20. Since I have an infection, I cannot get pregnant.	95.5%	93.6%	96.9%	0.007

p values were unadjusted by covariates. HPV=Human papillomavirus.

Supplementary Table S8. HPV-QoL questionnaire unadjusted scores by individual questions according to HPV vaccination status in the subsample of 1314 women with HPV infection (Vaccination = 759; No vaccination = 555).

		Mean	SD	SE	95% CI		p
					Lower limit	Upper limit	
Q1	Unvaccinated	2.64	1.21	.05	2.53	2.75	0.002
	Vaccinated	2.41	1.25	.05	2.32	2.50	
	Total	2.50	1.24	.04	2.43	2.57	
Q2	Unvaccinated	3.19	1.29	.06	3.08	3.31	0.932
	Vaccinated	3.19	1.28	.05	3.09	3.28	
	Total	3.19	1.28	.04	3.12	3.26	
Q3	Unvaccinated	2.56	1.25	.06	2.45	2.67	0.362
	Vaccinated	2.63	1.29	.05	2.54	2.72	
	Total	2.60	1.27	.04	2.53	2.67	
Q4	Unvaccinated	2.45	1.26	.06	2.34	2.56	0.629
	Vaccinated	2.42	1.25	.05	2.33	2.51	
	Total	2.43	1.25	.04	2.36	2.50	
Q5	Unvaccinated	4.06	1.04	.05	3.97	4.15	0.932
	Vaccinated	4.07	1.15	.04	3.98	4.15	
	Total	4.06	1.10	.03	4.00	4.13	
Q6	Unvaccinated	4.03	1.13	.05	3.93	4.13	0.417
	Vaccinated	3.98	1.21	.04	3.89	4.06	
	Total	4.00	1.18	.03	3.93	4.06	
Q7	Unvaccinated	1.90	1.10	.05	1.80	1.99	0.142
	Vaccinated	1.80	1.06	.04	1.73	1.88	
	Total	1.84	1.08	.03	1.78	1.90	
Q8	Unvaccinated	3.81	1.38	.06	3.69	3.94	0.044
	Vaccinated	3.98	1.36	.05	3.88	4.07	
	Total	3.91	1.37	.04	3.83	3.99	
Q9	Unvaccinated	1.63	.89	.04	1.55	1.71	0.539
	Vaccinated	1.66	.92	.03	1.59	1.73	
	Total	1.65	.90	.03	1.60	1.70	
Q10	Unvaccinated	1.70	.94	.04	1.61	1.78	0.335
	Vaccinated	1.64	.95	.04	1.58	1.71	
	Total	1.67	.94	.03	1.61	1.72	
Q11	Unvaccinated	1.88	1.07	.05	1.79	1.98	0.368
	Vaccinated	1.83	1.04	.04	1.75	1.90	
	Total	1.85	1.05	.03	1.79	1.91	
Q12	Unvaccinated	2.19	1.31	.06	2.08	2.31	0.309
	Vaccinated	2.12	1.22	.05	2.03	2.21	
	Total	2.15	1.26	.04	2.08	2.22	
Q13	Unvaccinated	2.83	1.47	.07	2.70	2.96	0.684
	Vaccinated	2.87	1.48	.05	2.76	2.97	
	Total	2.85	1.48	.04	2.77	2.93	
Q14	Unvaccinated	3.47	1.28	.06	3.36	3.59	0.377
	Vaccinated	3.54	1.31	.05	3.44	3.64	
	Total	3.51	1.30	.04	3.44	3.59	
Q15	Unvaccinated	3.38	1.38	.06	3.25	3.50	0.352
	Vaccinated	3.45	1.39	.05	3.35	3.55	
	Total	3.42	1.38	.04	3.34	3.50	

SD=Standard deviation; SE=Standard error; CI=Confidence interval; Q=question (item).

Supplementary Table S9. Missing data for each variable included in the study.

Variable	Missing (%)	Comments
Total evaluable sample: 1907 participants		
Nationality	0.0%	No missing data imputation
Age (years)	0.0%	No missing data imputation
BMI (Kg/m²)	10.5%	No missing data imputation
Educational background	2.7%	Missing imputed
Menopause status	0.9%	Missing imputed
Sexually active	1.2%	Missing imputed
Partner	0.2%	Missing imputed
Type of sexual partner	2.8%	Missing imputed
Number of partners	6.9%	Missing imputed
Age at first sexual intercourse	7.1%	Missing imputed
Gestations	1.0%	Missing imputed
Deliveries	1.0%	Missing imputed
Immunosuppression	2.9%	Missing imputed
Smoking	0.3%	Missing imputed
Alcohol	0.6%	Missing imputed
Exercise	0.3%	Missing imputed
Contraception	1.9%	Missing imputed
Type of Contraception	1.9%	Missing imputed
Previous STI	0.9%	Missing imputed
Cervical conization	4.3%	Missing imputed
Genital warts	4.9%	Missing imputed
Abnormal cytology	3.1%	Missing imputed
Information on HPV given by her doctor	35.3%	No missing data imputation
Vaccination data (valid sample for analysis of vaccinated participants: 930)		
Age (years) at vaccination	4.3%	Missing imputed
Type of HPV vaccine	33.3%	No missing data imputation
Number of doses administered	2.6%	Missing imputed
HPV after vaccination	66.2%	No missing data imputation
Type of HPV virus by risk after vaccination	70.9%	No missing data imputation
Cytology after vaccination	6.8%	Missing imputed
PROM (valid sample for analysis of PROMs: 1,314 HPV+)		
HAD scale (n=591)	55.0%	No missing data imputation
FSFI questionnaire (n=1,104)	16.0%	No missing data imputation
GHQ-12 questionnaire (n=1,185)	9.8%	Missing imputed
HPV-Know-Q questionnaire (n=1,279)	2.7%	Missing imputed
HPV-QoL questionnaire (n=1,225)	6.8%	Missing imputed

HPV = Human papilloma virus; HAD scale = Hospital anxiety and depression scale; FSFI = Female Sexual Function Index; GHQ-12 = Global health questionnaire-12 items; HPV-QoL = HPV-Quality-of-life questionnaire; BMI = Body Mass Index; STI = sexually transmitted infection.

Figure S1. Adjusted likelihood (percentage) of HPV vaccination according to age of women at first sexual relationship encounter, globally and by HPV infection status.

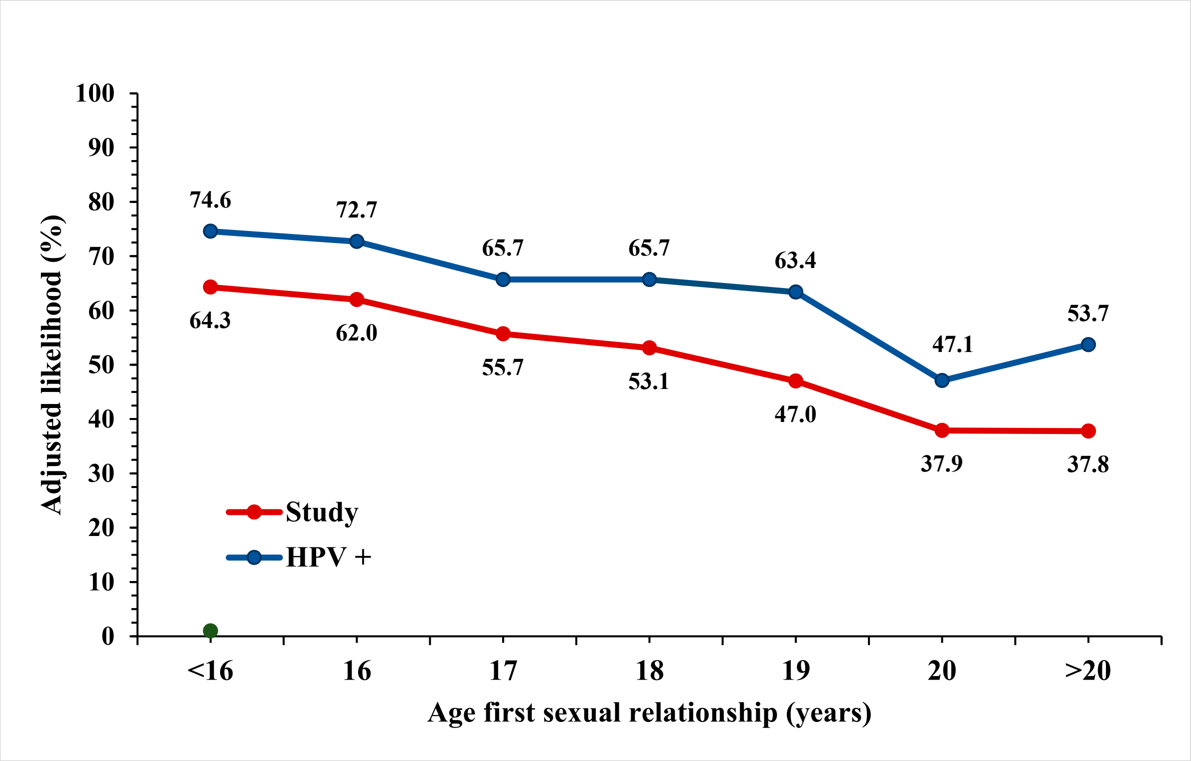

Lineal Chi^2: Study; 53.38, p<0.001, HPV+; 28.05, p<0.001.

Figure S2. Adjusted likelihood (percentage) of HPV vaccination according to number of sexual partners of participants' women, globally and by HPV infection status.

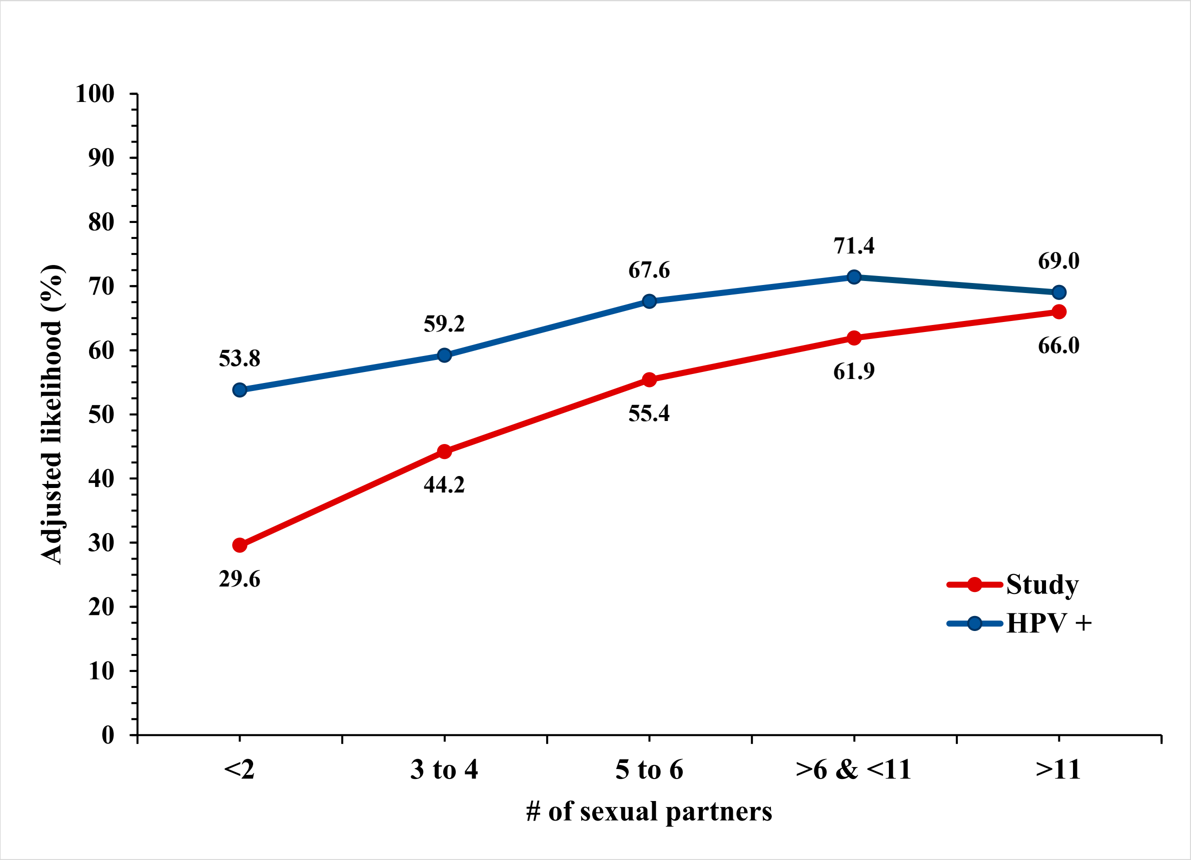

Lineal Chi^2: Study; 96.66, p<0.001, HPV+; 12.81, p<0.001.
